# Analysis of ocular emergencies in a reference eye center in Brazil

**DOI:** 10.5935/0004-2749.20220016

**Published:** 2025-08-21

**Authors:** Tiago de Assis Clemente de Araújo, Caio Rodrigo de Oliveira Melo, Larissa Paz Mendes, Taciana Mirely Maciel Higino, Camilla Silva da Rocha, Laura Patrícia Ferreira Sabino, Michel Bittencourt Santos

**Affiliations:** 1 Department of Ophthalmology, Fundação Altino Ventura, Recife, PE, Brazil; 2 Department of Scientific Investigation, Fundação Altino Ventura, Recife, PE, Brazil

**Keywords:** Emergency, Eye disease, Vision disorder, Conjunctivitis, Foreign body, Eye injury, Season, Brazil, Emergência, Oftalmopatia, Transtorno da visão, Conjuntivite, Corpo estranho, Traumatismo oculare, Estações do ano, Brasil

## Abstract

**Purpose:**

To determine the incidence of ocular emergencies and patient profiles in a public health eye center in Brazil.

**Methods:**

The medical record database of the Fundação Altino Ventura, Recife, Brazil was retrospectively analyzed and included all patients assisted at the ophthalmic emergency room between January 2017 and January 2018. Medical records with incomplete data and outpatient complaints were excluded. For records with multiple visits, only the initial visit was considered.

**Results:**

In 1 year, 134,788 patients (mean age at admission: 38.7 ± 22 years; range: 0-99 years) were admitted at the emergency room of the Fundação Altino Ventura. The most frequent diagnoses were conjunctivitis (52,732 cases; 37.3%), blepharitis (7,213 cases; 5.1%), and corneal/conjunctival foreign body (6,925 cases; 4.9%). Corneal/conjunctival foreign body and ocular trauma had an eightand two-fold higher incidence in male patients, respectively (both p<0.001). Female patients presented a two-fold higher incidence of trichiasis and blepharitis than males (p<0.001). Corneal/conjunctival foreign body and ocular trauma affected more patients in a productive age (>15 years), while corneal ulcers, blepharitis, and trichiasis affected more elderly patients. All diagnostic clusters (e.g., infectious diseases, ocular trauma, foreign bodies, retinopathies, eyelid disorders, corneal diseases, glaucomatous crisis, and neuroophthalmological diseases) were more common during the spring season (p<0.001).

**Conclusion:**

The most common ocular emergencies in the present study were infectious diseases and foreign body. However, the incidence of ophthalmological emergencies was influenced by the age and sex of patients, as well as the time of the year.

## Abstract (pt)

**Objetivo:**

Determinar a incidência de emergências oculares em um centro oftalmológico de referência no Brasil.

**Métodos:**

O banco de dados de prontuários da Fundação Altino Ventura, Recife, Brasil, foi analisado retrospectivamente e incluiu pacientes atendidos, entre janeiro de 2017 e janeiro de 2018, na sala de emergência oftalmológica. Foram excluídos os prontuários com dados incompletos e com quadros ambulatoriais. Apenas o primeiro atendimento na emergência foi considerado para análise.

**Resultados:**

Em um ano, 134.788 pacientes (idade média de 38,7 ± 22 anos [0-99 anos]) foram admitidos na emergência da Fundação Altino Ventura. Os diagnósticos mais frequentes foram conjuntivite (52.732 casos [37,3%]), blefarite (7.213 casos [5,1%]) e corpo estranho na córnea/conjuntiva (6.925 casos [4,9%]). Corpo estranho na córnea/conjuntiva e trauma ocular foram cerca de 8 vezes e 2 vezes mais incidente em indivíduos do sexo masculino, respectivamente (p<0,001 em ambos). Triquíase e blefarite afetaram ~2 vezes mais pacientes do sexo feminino, respectivamente (p<0,001 em ambos). Corpo estranho na córnea/conjuntiva e trauma ocular afetaram mais pacientes em idade produtiva (>15 anos), enquanto úlcera, blefarite e triquíase da córnea afetaram mais pacientes idosos. Todos os grupamentos de diagnóstico (doenças infecciosas, trauma ocular, corpos estranhos, retinopatias, doenças das pálpebras, doenças da córnea, crise glaucomatosa e doenças neurooftalmológicas) foram mais incidentes na primavera (valor de p<0,001).

**Conclusão:**

As emergências oftalmológicas mais comuns no presente estudo foram as doenças infecciosas e o corpo estranho. Porém, a incidência das emergências oculares são fluências pela faixa etária e gênero do paciente, além da época do ano.

## INTRODUCTION

Although the eyes constitute only 0.1% of the body surface, they are enormously important as they allow a more complete perception of the external environment through their functional differentiation^(1,2)^. Their small and delicate structure is considered vulnerable to external aggression and can be harmed by unexpected trauma^(3-6)^.

Ophthalmic emergencies correspond to 3% of the emergency room consultations in the United States of America, and approximately 13.6% of admissions in emergency services in Brazil^(7-9)^. Some ocular injuries are considered self-limit, whereas others, if not promptly treated, can lead to irreversible blindness. In the United States of America, 40,000-60,000 patients per year become blind due to trauma^(2)^.

Although ocular emergencies are not typically life-threatening, they inflict a heavy burden on health and the financial status of affected individuals. Therefore, understanding the profile and risk factors of patients is important in public health care to help elaborate prevention policies and improve medical services^(9,10)^.

The analysis of large datasets has enhanced disease prediction and prevention, supported evidence-based medical practice, and improved the quality and efficiency of healthcare service delivery^(10-12)^. The electronic health record datasets enable a better understanding of the demographic, behavioral, clinical decision, treatment, and clinical outcome profile of thousands of patients. In addition, a large number of variables allow more accurate and reliable association analysis^(13)^. Herein, we evaluated the most frequent causes of ophthalmological emergencies in a reference eye center in the Northeast region of Brazil.

## METHODS

In this retrospective study, the database of the Altino Ventura Foundation (Recife, Brazil) was used to retrieve the electronic medical data of patients admitted between January 2017 and January 2018 at the emergency room. The data were exported into an Excel^®^ spreadsheet (Microsoft Corp., Redmond, WA, USA) prior to analysis for better organization. Medical records with incomplete data and outpatient complaints were excluded. For patients with multiple visits, only the first visit was considered. The data collected from the medical records included age, sex, diagnosis, admission date, and city of origin.

The ocular emergencies were grouped into infectious diseases, ocular trauma, foreign bodies, retinopathies, eyelid disorders, corneal diseases, glaucomatous crisis, and neuroophthalmological diseases.

Qualitative variables are expressed by their absolute and relative frequencies. Quantitative variables are represented by their means. Correlation analyses between categorical variables were performed using the chi-squared test through the statistical program SPSS version 25.0 for Windows (IBM Corp., Armonk, NY, USA). For all conclusions, statistical significance was considered at the 5% level.

## RESULTS

This big data study included 134,788 patients admitted at the emergency room of the Fundação Altino Ventura over a period of one year. The mean age of patients at admission was 38.7 ± 22 years (range: 0-99 years), and 52,651 (39.1%) were adults aged 19-42 years. The sample included 69,917 males (51.9%) and 64,817 females (48.1%). The majority of patients (135,800 patients; 98.8%) were from the Pernambuco state (Table 1).

**Table 1:** Demographic data of patients assisted at the ophthalmological emergency room of the Altino Ventura Foundation between January 2017 and January 2018

Characteristics	Frequency
Sex	
Male	69,917(51.9%)
Female	64,871 (48.1%)
Residence	
Pernambuco state	133,230 (98.8%)
Other state	1,558 (1.2%)
Age	
<10 years	18,125 (13.4%)
11-18 years	7,001 (5.2%)
19-42 years	52,651 (39.0%)
43-58 years	28,456 (21.1%)
59-82 years	26,211 (19.4%)
>83 years	2,344 (1.7%)

The most commonly diagnosed ophthalmological emergencies were conjunctivitis (n=52,732; 37.3%), ble pharitis (n=7,213; 5.1%), and corneal foreign body (n=6,925; 4.9%) (Table 2).

**Table 2:** Main ophthalmological diagnoses observed in the ophthalmological emergency room of the Altino Ventura Foundation between January 2017 and January 2018

Diagnosis	Frequency
Conjunctivitis	52,732 (37.3%)
Blepharitis	7,213 (5.1%)
Corneal foreign body	6,925 (4.9%)
Hordeolus	6,454 (4.7%)
Corneal ulcer	5,202 (3.8%)
Keratitis	3,388 (2.5%)
Eye trauma	3,096 (2.2%)
Corneal abrasion	3,054 (2.2%)
Trichiasis	2,044 (1.5%)
Subconjunctival hemorrhage	1,735 (1.2%)
Retinal detachment	1,303 (0.9%)

Conjunctivitis was the most common infectious disease, accounting for 65.3% of the infectious disease admissions; retinal detachments were the most prevalent among the retinal diseases (28.7%); trichiasis was the most common eyelid disorder (56.4% of admissions); and diplopia (22.0%) was the most commonly reported symptom related to neuro-ophthalmic disorders.

Corneal/conjunctival foreign bodies (eight-fold) and ocular trauma (~two-fold) were more frequent in males than females (p<0.001). In contrast, blepharitis and trichiasis (~two-fold) were more commonly observed in females (p<0.001) (Table 3).

**Table 3:** Distribution of the ophthalmological diagnoses by sex at the ophthalmological emergency room of the Altino Ventura Foundation

Diagnosis	Sex				p-value^*^
	Female		Male		
	n	%	n	%	
Conjunctivitis	26,564	59.8%	26,168	53.1%	
Blepharitis	4,672	10.5%	2,541	5.2%	
Hordeolus	3,478	7.8%	2,976	6.0%	
Corneal ulcer	2,035	4.6%	3,167	6.4%	
Keratitis	1,631	3.7%	1,759	3.6%	
Trichiasis	1,446	3.3%	598	1.2%	**<0.001**
Corneal abrasion	1,202	2.7%	1,854	3.8%	
Subconjunctival hemorrhage	1,019	2.3%	716	1.5%	
Eye trauma	855	1.9%	2,241	4.5%	
Corneal foreign body	707	1.6%	6,219	12.6%	
Retinal detachment	522	1.2%	782	1.6%	
Glaucomatous crisis	255	0.6%	266	0.5%	

* Chi-squared test.

Blepharitis (~10-fold), trichiasis (151-fold), and corneal ulcer (~10-fold) were more common in elderly patients (p<0.001). Other emergencies, such as conjunctivitis (~three-fold) and hordeolum (~11-fold), were more prevalent in younger patients (p<0.001).

Some ocular diseases, such as foreign body in the cornea (~10-fold) and ocular trauma (~two-fold), had a higher incidence in the economically active population aged 15-65 years (p<0.001) (Table 4).

**Table 4:** Distribution of diagnoses by age group

Diagnosis	Age group								p-value^*^
	≤20 years		21–59 years		60–80 years		>80 years		
	n	%	n	%	n	%	n	%	
Conjunctivitis	16,527	72.3	31,130	56	4,606	34.3	469	26	
Blepharitis	492	2.2	3,771	6.8	2,534	18.5	416	23.1	
Corneal foreign body	489	2.1	5,898	10.6	512	3.8	27	1.5	
Retinal detachment	43	0.2	829	1.5	404	3.0	28	1.0	<0.001
Hordeolus	2,629	11.5	3,425	6.2	382	2.8	18	1.0	
Eye trauma	894	3.9	1,836	3.3	324	2.4	42	2.3	
Trichiasis	22	0.1	414	0.7	1,329	9.9	279	15.5	
Corneal ulcer	378	1.7	3,039	5.5	1,490	11.1	295	16.4	

* Chi-squared test

All diagnostic clusters were more prevalent during the spring season in Brazil (i.e., late September to late December) (p<0.001).

## DISCUSSION

Eye emergencies show a high incidence, and certain diagnoses are associated with a risk of poor visual prognosis. Therefore, these emergencies should be promptly addressed to prevent visual loss, which would impact the individual and the economy of the society^(1,2)^. Thus, identifying the main ophthalmic emergencies and comprehending the associated risks can prevent these events and improve medical assistance, particularly in big data ophthalmic research^(3,14,15)^.

Studies have reported that socioeconomic, demographic, and cultural characteristics, as well as the season of the year are the main factors influencing variations in the incidence and prognosis of emergency eye pathologies^(16-21)^. This statement is corroborated by the present study, since all diagnostic clusters in our sample were more prevalent during the spring season. A similar study in Turkey also showed a higher incidence of ophthalmic emergencies during spring. This observation was recorded despite the fact that Turkey has a temperate climate with greater temperature variations between seasons in contrast to the tropical climate of Pernambuco characterized by smaller variations^(7)^.

Regarding the epidemiological profile of the studied population, the present findings are consistent with those of a study conducted by Babineau and Sanchez. They reported a higher prevalence of males (52.5%) in ophthalmological emergency services and a higher number of consultations among young adults aged 2040 years (42.5%)^(13)^. These observations are attributed to the increased exposure of males to the environment and types of labor that increase the risk of infectious diseases and trauma^(9)^.

The study conducted by Pierre Filho et al. in northeastern Brazil showed that eye trauma was the major cause of emergency care^(15)^. However, similar to our results, Almeida et al. reported that conjunctivitis, corneal abrasions, corneal foreign body, and ocular trauma are the main causes of ophthalmological emergencies in the northeast region of Brazil^(8)^.

In the literature, conjunctivitis is the most common ocular infectious disease^(8)^. In addition, its incidence varies according to the age of patients and preferentially affects young individuals. This can be explained by the high infectivity of this pathology, which is closely associated with the lack of hygiene commonly noted in childhood^(22,23)^.

When assessing eyelid and eyelash diseases, there was also a higher incidence of infectious causes, including blepharitis. In addition to this infection, trichiasis was a frequent cause of urgent admission and both were more prevalent in females than males^(23)^. Fromstein et al. suggested that eyelid inflammation is more common in this population due to the intense manipulation and misuse of cosmetics^(24)^.

Other diagnoses, such as corneal foreign body and ocular trauma, were more frequent in males. Luo et al. reported similar results, which associated the high incidence of these conditions with the types of labor and exposure to trauma in this population^(25)^.This also elucidates the more common diagnoses in individuals in the productive phase of life. An exception is ocular trauma, which is also common in childhood and adolescence due to the lack of prophylaxis and protection of the eye in this age group^(26)^.

Other complaints and diagnoses, such as diplopia or retinal detachment, were also frequent and important causes of care for neuro-ophthalmic reasons or retinal changes^(27,28)^. Both of these conditions raise concern, as they can lead to poor visual prognosis^(27,29)^.

Herein, we demonstrated that analysis of large amounts of data can provide reliable and accurate information. Moreover, the importance of eye emergencies in the Brazilian social context, due to their high incidence, was also exhibited. Thus, based on these data, it was possible to determine the age and sex groups at higher risk for each emergency diagnosis, as well as predict the season of the year during which there should be an increase in the number of visits. In addition, this work enables specific public measures to be performed before an increase in the incidence of these conditions. This approach would help to reduce the number of visits and enable better assistance to patients.
